# Multipurpose Prevention Technologies: Biomedical Tools to Prevent HIV-1, HSV-2, and Unintended Pregnancies

**DOI:** 10.1155/2011/429403

**Published:** 2011-08-09

**Authors:** Andrea Ries Thurman, Meredith R. Clark, Gustavo F. Doncel

**Affiliations:** CONRAD Clinical Research Center, Eastern Virginia Medical School, 601 Colley Avenue, Norfolk, VA 23507, USA

## Abstract

Statistics clearly show an unmet need for highly effective contraception, especially in less developed countries. Many of these countries are at the core of the HIV/AIDS epidemic and show very high prevalence rates for other sexually transmitted infections (STIs) such as that caused by HSV-2. A woman at risk of unintended pregnancy due to unprotected intercourse is also at risk for HIV/STI. Owing to their causative interrelationship, combining protection against these conditions will result in enhanced prevention and health benefits. Existing multipurpose prevention modalities such as condoms and physical barriers, albeit efficacious, face cultural hurdles that have so far hindered their widespread use. Success has recently been demonstrated in large clinical trials, demonstrating proof of concept of microbicides in reducing the incidence of HIV-1 and HSV-2 among at-risk populations. The challenge heretofore is to refine these products to make them more potent, convenient, accessible, and acceptable. Potent antiviral drugs released topically in the female reproductive tract by innovative delivered systems and formulations will provide safe, effective, and acceptable multipurpose prevention tools. This paper provides an overview of existing and novel approaches to multipurpose prevention strategies.

## 1. Significant Need for Innovative, Effective, and Acceptable Multipurpose Prevention Technologies

Effective multipurpose prevention technologies (MPTs) for women's reproductive health is an area searching for innovative strategies and approaches to increase access and adherence [1]. Here, we will discuss technologies designed to prevent pregnancy and sexually transmitted infections (STIs) and combinations of antiviral drugs to provide synergistic prophylaxis against multiple STIs. Unintended or mistimed pregnancies and STIs are prevalent and morbid problems worldwide. Although distinct issues with varying causes, the same behavior, unprotected intercourse, puts a woman at risk for both problems. Therefore, prevention of two or more of these conditions could potentially be targeted by one multipurpose product.

## 2. Unintended or Mistimed Pregnancies: A Significant Problem Worldwide

Almost half of all pregnancies worldwide, estimated to be over 100 million annually, are unintended or mistimed [2, 3]. In 2008, this resulted in 43 million abortions, half of which were performed under unsafe conditions, leading to almost 100,000 maternal deaths and 5 million women left with temporary or permanent disabilities [3]. Despite the existence of a variety of effective contraceptives available worldwide, discontinuation or nonuse remains high, primarily due to cost, side effects, inconvenient dosing schedules, poor access to prescription products, and/or poor acceptance of the method. This results in an unacceptably high rate of unintended or mistimed pregnancies. Statistics clearly show an unmet need for highly effective contraception, especially in less developed countries, where 99% of maternal deaths occur [2, 3].

## 3. The Human Immunodeficiency Virus Type 1 (HIV-1) Pandemic

Less developed countries, especially those of sub-Saharan Africa and south Asia, are also at the core of the acquired immunodeficiency syndrome (AIDS) epidemic. Over 33 million people worldwide are infected with human immunodeficiency virus type 1 (HIV-1) and 22.4 million, the majority of whom are women, live in sub-Saharan Africa [4]. Although progress has been made in preventing new HIV-1 infections, AIDS-related illnesses remain a leading cause of death globally [5]. In 2008, some communities in the USA, including pockets of the District of Columbia, had HIV-1 incidence rates similar to that of sub-Saharan Africa [6].

## 4. Impact of Lower Genital Tract Infections on HIV-1 Acquisition

Despite a global pandemic, it is estimated that approximately 99% of unprotected vaginal exposures to HIV-1 do not result in a productive infection [7]. This is in part due to the formidable protection afforded by an intact, healthy cervicovaginal epithelium. Three lower genital tract infections have been highly associated with an increased susceptibility to HIV-1 infection, even after controlling for other high-risk sexual behaviors [8–10]. Specifically, they are *Trichomonas vaginalis* (TV) [11, 12], bacterial vaginosis (BV) [13–17], and herpes simplex virus type 2 (HSV-2) [18–20]. These infections are all highly prevalent, not reportable, frequently recurrent, and often asymptomatic. These attributes represent a perfect storm for perpetuating the spread of HIV-1. Sexual transmission of HIV-1, in the absence of cofactors, is poorly efficient. Lower genital tract infections enhance HIV-1 acquisition through the cervicovaginal mucosa by inducing a proinflammatory state, weakening the integrity of the epithelial barrier, and/or decreasing local innate immunity and normal epithelial defenses, in addition to disrupting the protective effects of the normal, lactobacilli-dominated vaginal microbiome [21]. This is why MPT products which protect against HIV-1 and lower genital tract infections which function as HIV-1 susceptibility cofactors are likely to have a synergistic effect in stemming the epidemic.

## 5. Rationale for Multipurpose Prevention Technologies (MPTs)

MPTs offer solutions to more than one reproductive need. This translates into several benefits for the users such as improved convenience, adherence, effectiveness, cost-reduction, and environmental impact. For the most part, existing MPTs, for example, male condoms, have been proven to be effective against unintended pregnancies and STIs [22, 23]. However, prevalence of use is generally low due to acceptance and compliance issues [24]. New MPTs should be more effective and less user dependent, not interfere with sexual pleasure, and provide additional health benefits.

## 6. Existing MPTs and Improved Derivatives

### 6.1. Male and Female Condoms

MPT barrier methods include the male and female condom, diaphragms, and cervical caps. The cornerstone MPT is the male condom. This established product provides highly effective protection from pregnancy [22] and HIV-1 [23], when used correctly. Low cost and worldwide over-the-counter availability are additional desirable features of the male condom. The main obstacle to using the male condom as an effective MPT, however, is reflected in the disparity between the theoretical failure rates (2-3%) versus typical failure rates at one year of use (approximately 15%), indicating that this method suffers from inconsistent and improper use, mainly due to acceptability issues [24]. Many women cannot negotiate condom use with their male partner, as this could imply distrust in the relationship, so many experts advocate considering other options for MPT [25]. Health and community education has been a main format of increasing acceptance of condoms, particularly by men [25]. Access to free male condoms has reduced the incidence of STIs in targeted populations, such as military members [26]. Other strategies such as advocating for reduced pricing of condoms or distributing free condom packs at nightclubs frequented by at-risk groups, such as men who have sex with men, have also been undertaken to reduce the spread of STIs [27].

**Figure 1:**
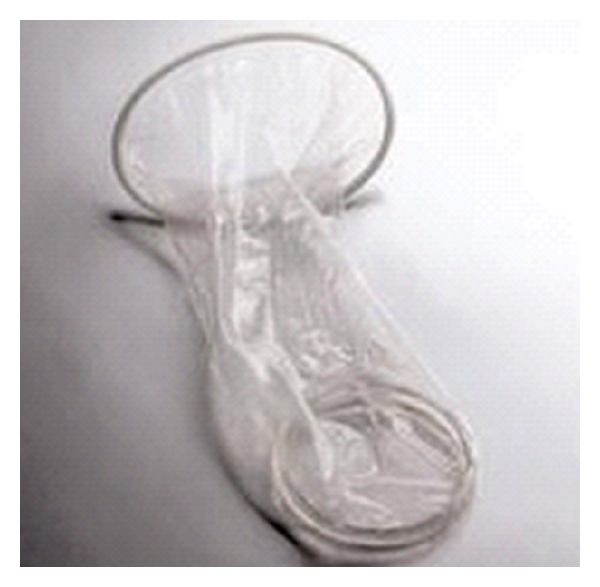
Design of the FC1 and the FC2.

The female condom (FC) has been advocated as a means for a female controlled physical barrier method to prevent pregnancy and STIs. Female condoms, like male condoms, prevent semen from reaching the cervix and vagina. The contraceptive efficacy of female condoms has been shown to be comparable to or even slightly higher than male condoms [28, 29]. The female condom performed as well as the male condom in reducing the recurrence of bacterial STIs [30]. Although there are no formal HIV-1 prevention trials with the female condom, mathematical models predict an effectiveness of 63–82% [31, 32]. The first female condom or FC1 was approved by the United States (US) Food and Drug Administration (FDA) in 1993. It is made of polyurethane and marketed under many trade names including the Reality female condom or the FC1 female condom [33, 34]. However, the polyurethane made a distracting crinkling noise with intercourse and was more expensive than the FC2, a newer female condom, manufactured by the Female Health Company, which is made of a synthetic latex (nitrile) [34]. The cost of the FC2 remains higher than that of a male condom. In an effort to further reduce costs and improve acceptability, organizations such as the World Health Organization (WHO) have investigated the performance of the FC2 using an *in vitro* design, after subjecting the condoms to multiple rounds of washing, disinfecting, drying, and relubrication, and found that the integrity of the condoms remained high [35]. Although the FC2 is currently recommended for single use, epidemiologic studies show the FC2 to have comparable cost-effectiveness to the male condom, especially when widely distributed to at-risk populations as a method to decrease HIV-1 transmission [32]. The FC1 and FC2 condoms are shown in Figure 1.

**Figure 2:**
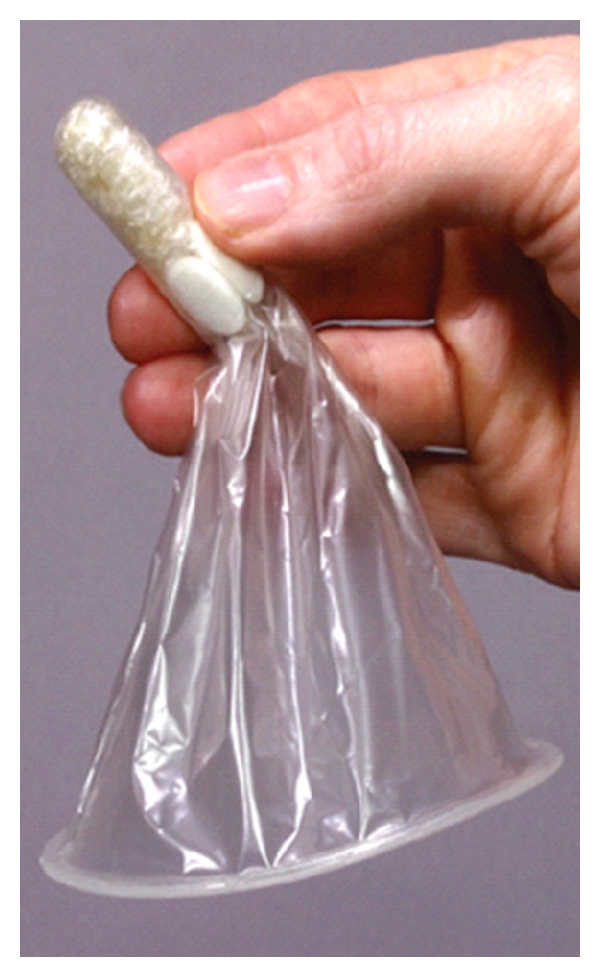
PATH Woman's Condom.

In an effort to increase acceptance and use of the female condom, the Program for Appropriate Technology in Health (PATH) has produced a novel FC product, the contraceptive efficacy of which is currently being tested in an NICHD-funded multicenter trial. A subset study, conducted by CONRAD, will look at biomarkers of semen exposure in the female vagina, before and after product use. The PATH Woman's Condom (WC) performed well in a short-term acceptability study and in a comparative crossover study with the FC1 [36, 37]. The WC has a pliable polyurethane pouch, a soft outer ring, and 4 elliptical foam pieces on the outside of the pouch that cling to the vagina to stabilize the device. The distal end of the pouch and foam pieces are packaged in a capsule that serves as an insertion aid and dissolves quickly after insertion (Figure 2). This design change was put in place to increase acceptability of the WC.

### 6.2. Female Diaphragms

The cervix has a higher density of CD4 cells and CCR5 chemokine receptors than the vagina [38]. It has also been identified as an initial site of SIV infection in macaque experiments [39]. These and other lines of evidence suggest that the endocervix is a primary infection site for HIV-1 and other STIs [40]. Several groups have tried to employ diaphragms as MPTs. Diaphragms have traditionally been used for contraception and offer similar failure rates as male condoms [41]. Cross-sectional studies and case control studies showed that acquisition of *Neisseria gonorrhoeae *(GC) and *Chlamydia trachomatis* (CT) is lower among women who choose diaphragms than women who choose other nonbarrier methods of contraception [42, 43] reviewed in [44]. Based on these observational data, the Methods for Improving Reproductive Health in Africa or MIRA trial was launched to compare the efficacy of the Ortho All-Flex diaphragm, lubricant gel (Replens) and condoms to condoms alone in preventing HIV-1, GC, and CT in at-risk women [45, 46]. In these large trials, there was no statistically significant difference in the incidence of HIV-1 [45] or GC and CT [46] among the two cohorts. However, the proportion of women using condoms in the diaphragm plus condom group was significantly lower than the condom alone group (54% versus 85%, *P* < 0.001), suggesting that the diaphragm plus lubricant provided at least as effective protection against HIV-1, GC, and CT as condoms alone, although these studies were not designed to test a noninferiority hypothesis [45, 46].

**Figure 3:**
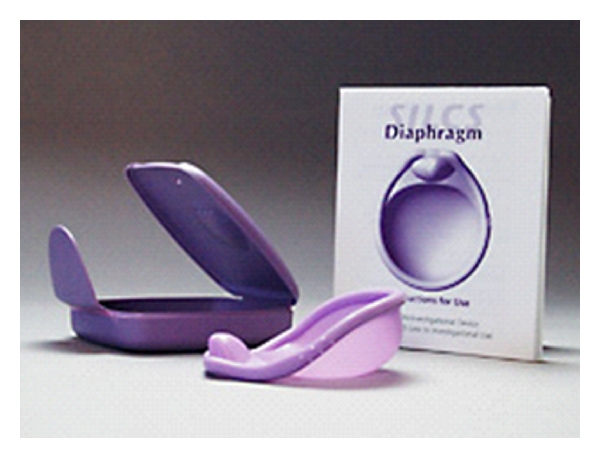
The SILCS diaphragm.

A disadvantage of traditional female diaphragms is that they require fitting by a health care professional and are therefore available by prescription only in many countries. PATH, in collaboration with CONRAD, developed and tested the SILCS diaphragm, a new, single size (one size fits most) contraceptive diaphragm designed to offer easier insertion and removal, increased comfort, elimination of latex-related odors and allergic reactions, and greater durability than latex diaphragms. The SILCS diaphragm is made of silicone, which is sturdy and more tolerant of extreme storage temperatures. It has a polymer material spring, which is less expensive and easier to assemble than metal springs, which are used in several latex diaphragms. The SILCS diaphragm performed well in phase I postcoital barrier effectiveness testing, performed in the USA [47]. Data also showed that couples in low resource settings preferred the SILCS diaphragm over the Ortho All-Flex diaphragm in a comparative cross-over study [48]. CONRAD studied the clinical efficacy of the SILCS diaphragm with BufferGel, from 2008 to 2009 in a USAID-funded study. The data from this study are in analysis, and FDA approval of this device could be as early as this year. The SILCS diaphragm (shown in Figure 3) is currently also being considered as a delivery system for tenofovir 1% gel and other candidate microbicides.

### 6.3. Chemical Barriers (Contraceptive Microbicides)

Spermicides are the mainstay of contraceptive chemical barrier methods. In the USA, the only FDA-approved spermicide available, which is widely used as a condom lubricant or in vaginally inserted foams, gels, and films, is nonoxynol-9 (N9). N9 is a surfactant that immobilizes or kills sperm by disrupting the lipid membrane. Early in the development of dual technology products, N9 and C31G (also known as SAVVY, developed by BioSyn, Inc.), both surfactants, were found to inhibit HIV-1 cell entry *in vitro *[49–53]. However, the *in vitro* and early preclinical and clinical data did not translate into effective, large-scale *in vivo* prevention methods, with some data indicating that frequent use of surfactants increased the susceptibility of the female genital tract to HIV-1, likely due to subclinical inflammation caused by these detergents [54–56].

The second generation of chemical barriers as MPTs was large, polyanionic sulfated, or sulfonated polymers that targeted the positively charged regions of the viral envelope of HIV-1 and/or blocked attachment, fusion or entry of the virus into host cells. These compounds included cellulose sulfate (CS or Ushercell, developed by TOPCAD/CONRAD), Carrageenan (also known as Carraguard and PC-515, developed by the Population Council), and PRO 2000 (also known as PRO 2000/5, developed by Indevus Pharmaceuticals). Ushercell also showed contraceptive properties equivalent to that of N9, in a noncomparative clinical trial where a 6% CS gel was used by 200 couples as their sole contraceptive method [57]. Although *in vitro* and early clinical data were promising for all these different HIV-1 entry inhibitors, large-scale prevention trials showed no increased protection over placebo [58–61].

Finally, products which worked to maintain the normal acidic pH of the vagina were introduced as potential MPTs. It was hypothesized that these products would maintain the healthy vaginal pH in the presence of semen and would work to partially inactivate pathogens that are acid sensitive, including HIV-1. Unfortunately, BufferGel (developed by ReProtect LLC) was found to have no significant protective effect for HIV-1 [62]. However, it was shown to be safe and spermicidal [63].  Table 1 summarizes recent MPT clinical trials.

Although research and development of dual contraceptive microbicides has yet to produce a safe and effective product, researchers continue to discover new approaches to achieve this goal. Examples of these efforts include dermaseptins, magainins and other antimicrobial peptides [68–71], AZT derivatives [72], cellulose conjugates [73], and other new dual action compounds [68].

## 7. Innovative MPTs on the Horizon

Results from the randomized, double-blind, placebo-controlled Centre for the AIDS Program of Research in South Africa (CAPRISA) 004 trial, demonstrating that 1% tenofovir vaginal gel reduced the incidence of HIV-1 by 39% overall and also reduced the incidence of primary HSV-2 infection by 51%, provided proof that potent antiretroviral agents could prevent HIV-1 and other prevalent HIV-1 susceptibility cofactors like HSV-2 [74]. Although a resounding proof-of-concept, the observed 39% protection certainly leaves room for improvement. Reduced user-dependence, more convenient dosing, and increased potency are some of the goals for improving future MPTs.

### 7.1. Innovative Dosage Forms for Intravaginal Delivery of Tenofovir for the Prevention of HIV-1 and HSV1/2 Sexual Transmission

Adherence is a prime concern in large prevention trials. Although all users of tenofovir gel experienced a significant protection against HIV-1 acquisition in the CAPRISA 004 trial, protection was related to adherence. Specifically, high (>80% of sexual acts protected by microbicide use), intermediate (50–80% of sexual acts used microbicide), and low adherence (<50% of sexual acts associated with microbicide use) users had a 54%, 38%, and 28% reduction in HIV-1 incidence, respectively, compared to placebo users [74]. To increase adherence, CONRAD is currently developing an intravaginal ring capable of releasing tenofovir in amounts that generate tissue concentrations similar to those attained by the 1% gel in a rabbit pharmacokinetic (PK) study. Designed in collaboration with Dr. Kiser (University of Utah), the tenofovir ring is made of hydrophilic polyurethane loaded with more than 1 gram of tenofovir, with a daily release rate of at least 10 mg/d for a target 90-day duration. The ring has approximate dimensions of 55 mm outer diameter and 5.5 mm cross-sectional diameter, only slightly larger than the commercially available contraceptive NuvaRing. Phase 1 clinical evaluation of the tenofovir ring is expected to initiate in early 2012.

Other intravaginal dosage forms for TFV are a fast-dissolve film and a fast-dissolve tablet. The TFV fast dissolve film, under development at the University of Pittsburgh and Magee-Womens Research Institute, is also small and is expected to provide similar tissue concentrations of TFV as compared to the TFV gel given its concentrated TFV dose. The tablet is under development by CONRAD with an estimated phase I trial in 2012. Unlike the gel dosage form, use of a vaginal applicator is not required for the film or the tablet and multiple doses may be packaged together in compact containers, thereby making them attractive dosage forms based on increased portability and potentially lowering manufacturing costs and environmental impact.

### 7.2. Enhanced Protection against HSV/HIV Genital Infection

Multiple lines of evidence indicate that HSV-2 infection is a significant HIV-1 susceptibility cofactor and that HSV-2 and HIV-1 have potential negative impacts on both infections [18, 19, 75–78]. However, despite the strong epidemiologic and observational data linking these two infections, randomized controlled trials using prophylactic oral acyclovir (ACV) in HSV-2-positive individuals to reduce the incidence of HIV-1 have not shown a significant preventative effect [79, 80]. In addition, ACV prophylaxis in HSV-2 and HIV-1 seropositive individuals did not reduce transmission of HIV-1 to seronegative sexual partners [81]. The potential reasons for failure of the interventional trials are several but include persistence of inflammatory infiltrates, compliance with prophylactic therapy, and adequate delivery of antiviral medications to genital tissues.

Frank disruption of the genital epithelium by HSV-2 lesions is likely not the only mechanism by which the genital mucosa becomes more susceptible to HIV-1 infection [21]. The surrounding epithelium has subclinical changes in inflammatory and innate immune response, including an influx of CD4+ and CD8+ cells, which persist for months even after an epithelial lesion has healed, leaving apparently normal genital epithelium still vulnerable to HIV-1 infection [82, 83]. Oral ACV therapy does not alter the persistence of these HIV-1 target cells [82, 83]. 

Compliance with prophylactic therapy was assessed by participant report and pill counting [80] and with serum and urine ACV levels [79]. PK data indicated that compliance with daily oral therapy was poor in one trial [79]. It is likely that compliance with preventative treatment would increase with a more convenient and long acting dosing regimen such as an intravaginal ring. It is known that plasma and vaginal levels of ACV are poorly correlated (*r* = 0.28, *P* > 0.05), with vaginal secretions containing 15–170% (mean 79%) of plasma ACV levels [84, 85]. PK studies demonstrate that peak vaginal concentrations of ACV are reached 30–60 minutes after oral dosing [85]. Peak vaginal concentrations of ACV (0.8–3.6 nmols/g or 0.18–0.81 *μ*g/mL) after daily oral dosing have also been shown to be below the 50% inhibitory dose of ACV for HSV-2 (approximately 0.91 *μ*g/mL) [86]. These data suggest that higher oral doses of ACV would be required to prevent primary infection by HSV-2 or to reduce recurrent genital epithelial replication and shedding. In addition, missed doses of oral ACV may significantly affect adequate genital concentrations of the drug.

This is why we have begun to develop a tenofovir and ACV combination vaginal product. We believe that the efficacy of tenofovir in preventing primary HSV-2 infections, shown in the CAPRISA 004 study [74], may be improved significantly by adding topical delivery of ACV. We also hypothesize that increasing the concentration of tenofovir in the genital tissues will increase its effectiveness. These hypotheses are supported by data from the CAPRISA-004 trial, which directly correlated the concentrations of tenofovir and its active metabolite tenofovir diphosphate, in genital tract secretions and tissues with the likelihood of preventing HIV-1 or HSV-2 [87]. Tenofovir and ACV will be synergistic in an MPT product to prevent HIV-1 and HSV-2. We hypothesize that ACV, delivered topically and vaginally, will result in higher local tissue concentrations, which would reduce or control HSV-2 epithelial replication and the subclinical changes in the genital mucosa associated with recurrent HSV-2 outbreaks.

CONRAD is currently developing an intravaginal ring for the sustained dual delivery of tenofovir and ACV. Tenofovir and ACV have similar hydrophilicity and therefore may be suitably coformulated in hydrophilic polyurethanes. In collaboration with Controlled Therapeutics (East Kilbride, Scotland), a 10% tenofovir/10% ACV hydrophilic polyurethane ring with dimensions similar to NuvaRing was developed that is capable of releasing milligram quantities of each drug daily for up to one month. Ongoing product development efforts continue to increase these release rates as well as extend the duration of drug release.

Another intravaginal ring design well suited for use as a sustained MPT dosage form, and particularly for codelivery of tenofovir and ACV, is the drug pod-based Versaring technology currently being developed by Auritec and Oak Crest Research Institute (Pasadena, Calif). This technology uses a silicone elastomer ring as primarily a physical holder for ten individually formulated—and subsequently independently rate-controlled—drug cores or pods (~3–20 mg drug per pod) that provides remarkably controlled release profiles. With CONRAD's collaboration, a tenofovir/ACV combination intravaginal ring using this technology is also currently under development.

Although randomized controlled trials of Carraguard failed to demonstrate efficacy in preventing HIV-1 infection [59], it was found to be safe, well tolerated, and physically stable. These properties make carrageenan a good delivery vehicle for other microbicide candidates. Furthermore, it has demonstrated high anti-HPV activity *in vitro* [88]. The Population Council commenced early preclinical and animal testing of a combination MIV-150, a nonnucleoside reverse transcriptase inhibitor developed by Medivir and zinc acetate, prepared in a delivery vehicle of carrageenan gel [89], named PC 1005. This MPT showed complete protection against RT-SHIV infection for up to 8 hours after daily dosing for 14 days [89]. *In vitro* data suggest that zinc salts have activity against HIV-1 [90] and HSV-2 [91, 92].

### 7.3. Innovative MPT for the Prevention of HIV-1 Acquisition and Unintended Pregnancies

**Figure 4:**
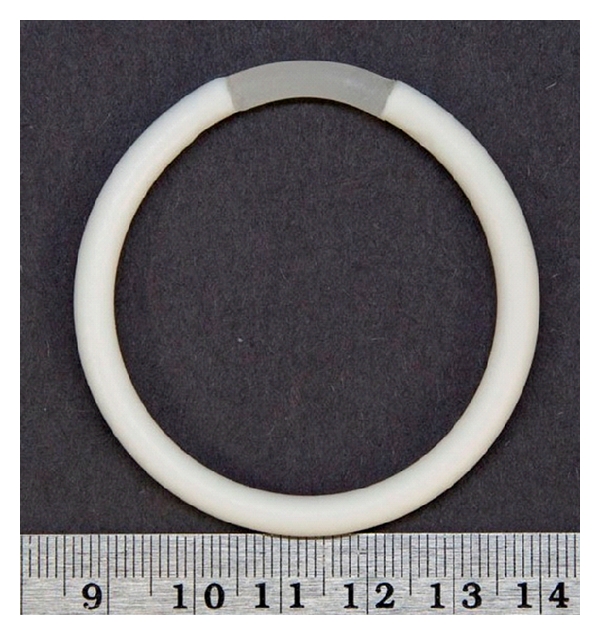
Segmented intravaginal ring for codelivery of tenofovir (long white segment) and levonorgestrel (short segment). Photo courtesy of Dr. Patrick Kiser (University of Utah).

Although the recent news of tenofovir as an anti-HIV-1 microbicide is compelling, tenofovir is not a contraceptive. To solve this problem, we are also currently developing an MPT with extended dosing of levonorgestrel (LNG) and tenofovir in an intravaginal ring which would be effective for at least 90 days (Figure 4). We chose LNG because of its long track record of safety and efficacy [93, 94]. The WHO previously developed a microdose LNG eluting silicone vaginal ring as a one-year contraceptive product, which went through extensive safety, efficacy, and acceptability testing. The product was not pursued at the time, however, due to funding considerations [95–97]. 

The tenofovir/LNG intravaginal ring under development in collaboration with the University of Utah builds on both the millidose tenofovir vaginal ring described above and WHO's work on the microdose LNG ring. One of the major challenges to developing a combination ring for these two particular drugs is the large (three orders of magnitude) difference in target release rates between tenofovir and LNG. Moreover, LNG's hydrophobicity lends itself to formulation in, and delivery from, hydrophobic polymers. The design of our ring overcomes both of these challenges by formulating the drugs separately in two independent drug-loaded segments, with the tenofovir segment comprising more than 80% of the ring's total volume (maintaining the ring's capacity to deliver 10 mg/d tenofovir) and the short LNG segment formulated with hydrophobic polyurethanes for delivery of 20 *μ*g/d LNG. Dr. Kiser's group has previously reported on two-segment polyurethane intravaginal rings for the codelivery of tenofovir and dapivirine [98]. Phase 2b dose and safety testing of this product is expected to begin in the next year.

In collaboration with PATH (Program for Appropriate Technology in Health), CONRAD is also testing the delivery of TFV gel by SILCS, a one-size-fits-all diaphragm with contraceptive barrier properties. This combination should provide coitally associated contraception and prevention of HIV-1 and HSV-2 acquisition.

The International Partnership for Microbicides (IPM) has developed an intravaginal ring which elutes dapivirine (TMC120), a nonnucleoside reverse transcriptase inhibitor (NNRTI), for one month [99–101]. Dapivirine has been shown to be effective in preventing HIV-1 infection *in vitro*, using cell line and tissue explant models [102]. Dapivirine has been studied in phase 1 and 2 dosing and safety studies [103, 104]. IPM has partnered with the Population Council in developing an MPT intravaginal ring which elutes dapivirine and levonorgestrel, showing promise in early laboratory and preclinical studies [105].

## 8. Conclusions

Multipurpose prevention technologies, MPTs, encompass simultaneous prevention of STIs and unintended pregnancies as well as prevention of more than one STI or reproductive tract infection. Established methods include barrier devices such as male and female condoms and female diaphragms. Innovations to these traditional MPT products are underway to improve accessibility, acceptability, and efficacy. Although clinical trials failed to demonstrate efficacy against HIV-1 transmission for the first generations of chemical MPT products including spermicides (nonoxynol-9 and C31G), polyanionic sulfated or sulfonated polymers (cellulose sulfate, carrageenan, and PRO 2000), and vaginal pH buffers (BufferGel), research and development continues to optimize the contraceptive properties of some of these compounds or to use these compounds as delivery vehicles for more potent antivirals or spermicides. Some of them, for instance carrageenan, are being further considered for new applications. Proof of concept for a chemical MPT, tenofovir vaginal gel, has just been demonstrated for the prevention of HIV-1 and primary HSV-2 infections. Research is underway to simplify the dosing of tenofovir vaginal gel and develop other delivery systems for tenofovir alone or in combination with contraceptive hormones (e.g., levonorgestrel) or other potent antivirals.

Women at risk for unintended pregnancies are by definition also at risk of STIs, and women who are at risk of one STI are also likely at risk of several different STIs. Data support that women find MPT products desirable and acceptable [106]. Simplifying and extending the dosing regimen, decoupling prophylaxis from coital acts, and developing female controlled protection products are all innovative strategies and approaches to increase the use of these much needed technologies. MPTs are likely to have a synergistic impact on the epidemics of sexually transmitted infections and unintended pregnancies and facilitate implementation and uptake of these biomedical preventative interventions.

## Figures and Tables

**Table 1:** Contraceptive and microbicide data on multipurpose prevention technologies.

Product/developer (common brands)	*In vivo* human contraceptive data	*In vivo* human microbicide data	
	Supportive	Supportive	Non-supportive/Inconclusive
Physical barriers			

Male condom/several	*√* [22]	*√* [23]	
Female condom/several (FC1, FC2, PATH Women's Condom)	*√* [28, 29]	*√* [30–32]	
Female diaphragm/several (Ortho All Flex)	*√* [41]		*√* [45, 46]
SILCS diaphragm/CONRAD	Data in Analysis	Not studied	

chemical Barriers: surfactants			

Nonoxynol-9/several	*√*		*√* [55]
C31G/BioSyn (SAVVY)	*√* [64]		*√* [54, 56]

Chemical barriers: attachment, entry and fusion inhibitors: (polyanionic sulfated or sulfonated polymers)			

Cellulose Sulfate (Ushercell, CS)/Polydex Pharmaceuticals, Program for the Topical Prevention of Conception and Disease (Rush University), CONRAD	*√* [57]		*√* [58, 61]
Carrageenan/Population Council (Carraguard, PC-515)			*√* [59]
PRO2000/Indevus Pharmaceutical (PRO2000/5)	*In vitro*, no large human studies		*√* [60, 62]

Acid buffers and vaginal defense enhancers			

BufferGel/ReProtect LLC	*√* [65]		*√* [62]
Acidform Gel (Amphora) Instead Sciences	*√* [66, 67]	No large human efficacy trials	
